# LOCALIZED FLEXURAL BULLOUS PEMPHIGOID

**DOI:** 10.4103/0019-5154.43214

**Published:** 2008

**Authors:** Vandana Mehta, C Balachandran

**Affiliations:** *From the Department of Skin and STD, Kasturba Medical College, Manipal, Karnataka, India. Email: vandanamht@yahoo.com*

A 63-year-old male agriculturist presented with a spontaneously appearing bullous eruption localized to the axillae and groins, with intense pruritus of 3 weeks duration. He was also a known hypertensive, diagnosed with renal failure on treatment with ACE inhibitors and amlodepin since 1 year. On examination there were tense vesicles and bullae associated with crusting and erosions in the axillae (Fig. 1), upper medial aspect of arms and groins bilaterally (Fig. 2). The rest of the skin including the mucous membranes, palms, soles, genitalia were normal. Histopathology revealed subepidermal blisters, direct immunofluorescence assay of perilesional skin showed linear deposits of IgG and C3 along the basement membrane zone and the indirect immunofluorescence demonstrated antibasement membrane antibodies bound to the epidermal side of the salt split normal human skin thus confirming our diagnosis of localized bullous pemphigoid. Patient was accordingly started on prednisolone 60 mg daily in tapering doses which resulted in complete healing of the erosions within a month.

**Fig. 1:**
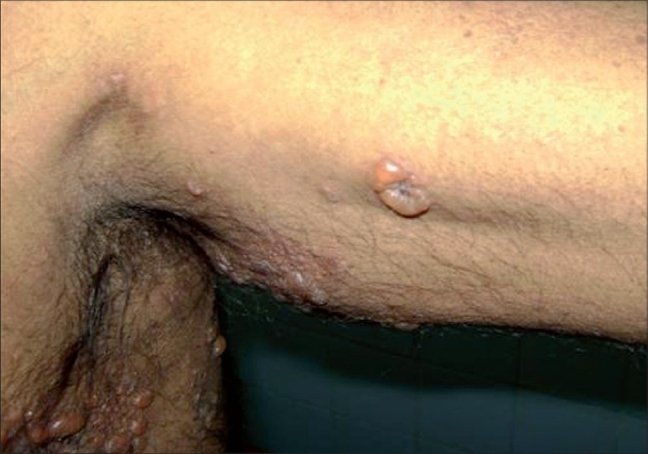
Tense vesicles and bullae in the axilla

**Fig. 2:**
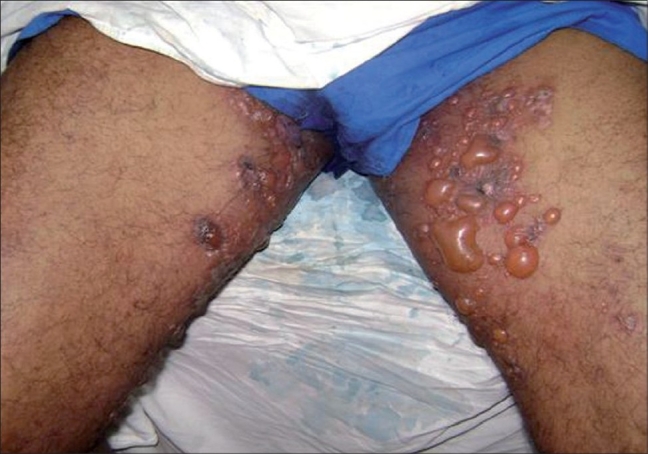
Tense vesicles and bullae in the groins

Localized bullous pemphigoid (LBP) is a rare autoimmune subepidermal blistering disease of the elderly characterized by chronic intermittent eruptions affecting only a restricted area of the body. Though it accounts for 16% to 29% of all cases of bullous pemphigoid, the true incidence may be greater as it is often misdiagnosed and is highly responsive to topical steroids. LBP has got similar clinical, histopathological and immunofluorescence features to generalized bullous pemphigoid.^1^ Three types have been identified which include: 1) mucous membrane pemphigoid or cicatricial pemphigoid 2) localized scarring pemphigoid or Brunsting Perry pemphigoid affecting the head and neck 3) localized non scarring pemphigoid usually seen over the pretibial region, vulva, breast and the soles. The diagnosis of this last entity tends to be delayed because it can mimic other localized vesicobullous diseases and dyshidrotic eczema.^2^ While the pathogenesis of generalized bullous pemphigoid is well elucidated, it is unknown why patients with LBP have limited disease. The pathogenesis most likely could be similar to that of generalized bullous pemphigoid because patients in both the groups recognize the same BP antigens.^3^ LBP has been documented following radiotherapy,^4^ PUVA therapy, trauma,^5^ sunexposure,^6^ split skin grafting for burns,^7^ around peristomal lesions^8^ and several authors have thus postulated that these local factors might play a role in the induction of lesions in immunologically susceptible individuals. In a study on the distribution of bullous pemphigoid antigens in normal human skin the greatest expression was seen in the skin obtained from the flexor aspect of arms, legs and thighs^9^ which probably explains the predominant flexural localization of lesions in our case. Whether the ACE inhibitors contributed in triggering the bullous eruption here is not clear as the patient had been taking the above medications for almost a year. Nevertheless this case emphasizes the need to follow-up such patients regularly as they are at risk of developing a generalized eruption later in life.

## References

[1] Tran JT, Mutasim DF (2005). Localized bullous pemphigoid: A commonly delayed diagnosis. Int J Dermatol.

[2] Scola F, Telang GH, Swartz C (1995). Dyshidrosiform pemphigoid. J Am Acad Dermatol.

[3] Soh H, Hosokawa H, Miyauchi H, Izumi H, Asada Y (1991). Localized bullous pemphigoid shares the same target antigen as bullous pemphigoid. Br J Dermatol.

[4] Leconte-Boulard C, Dompmartin A, Verneuil L, Thomine E, Joly P, Rogerie MJ (2000). Localized bullous pemphigoid following radiotherapy. Ann Dermatol Venereol.

[5] Vermeulen C, Janier M, Panse I, Daniel F (2000). Localized bullous pemphigoid induced by thermal burn. Ann Dermatol Venereol.

[6] Lee CW, Ro YS (1992). Sun induced localized bullous pemphigoid. Br J Dermatol.

[7] Hafejee A, Coulson IH (2005). Localized bullous pemphigoid 20 years after split skin grafting. Clin Exp Dermatol.

[8] Torchia D, Caproni M, Ketabchi S, Antiga E, Fabbri P (2006). Bullous pemphigoid initially localized around a urostomy. Int J Dermatol.

[9] Hamm G, Wozniak KD (1988). Bullous pemphigoid antigen concentration in normal skin: relation to body area and age. Arch Dermatol Res.

